# Immunogenicity and Cross-Protective Efficacy Induced by Outer Membrane Proteins from *Salmonella* Typhimurium Mutants with Truncated LPS in Mice

**DOI:** 10.3390/ijms17030416

**Published:** 2016-03-22

**Authors:** Qiong Liu, Qing Liu, Xinxin Zhao, Tian Liu, Jie Yi, Kang Liang, Qingke Kong

**Affiliations:** 1 Institute of Preventive Veterinary Medicine, Sichuan Agricultural University, Chengdu 611130, China; p19890528@126.com (Q.L.); xxinzhao@163.com (X.Z.); lt9208101223@163.com (T.L.); jelly187@163.com (J.Y.); LKang024@163.com (K.L.); 2 Department of Bioengineering, College of Veterinary Medicine, Sichuan Agricultural University, Chengdu 611130, China

**Keywords:** lipopolysaccharide, outer membrane proteins, cross-protection

## Abstract

Lipopolysaccharide (LPS) is a major virulence factor present in the outer membrane of *Salmonella enterica* serovar Typhimurium (*S.* Typhimurium). Outer membrane proteins (OMPs) from *Salmonella* show high immunogenicity and provide protection against *Salmonella* infection, and truncated LPS alters the outer membrane composition of the cell wall. In our previous study, we demonstrated that *Salmonella* mutants carrying truncated LPS failed to induce strong immune responses and cross-reaction to other enteric bacteria, due to their high attenuation and low colonization in the host. Therefore, we plan to investigate whether outer membrane proteins from *Salmonella* mutants with truncated LPS resulting from a series of nonpolar mutations, including ∆*waaC12*, ∆*waaF15*, ∆*waaG42*, ∆*rfaH49*, ∆*waaI43*, ∆*waaJ44*, ∆*waaL46*, ∆*wbaP45* and ∆*wzy-48*, affect immunogenicity and provide protection against diverse *Salmonella* challenge. In this study, the immunogenicity and cross-protection efficiency of purified OMPs from all mutants were investigated to explore a potential OMP vaccine to protect against homologous or heterologous serotype *Salmonella* challenge. The results demonstrated that OMPs from three *Salmonella* mutants (∆*waaC12*, ∆*waaJ44* and ∆*waaL46*) induced higher immune responses and provided good protection against homologous *S.* Typhimurium. The OMPs from these three mutants were also selected to determine the cross-protective efficacy against homologous and heterologous serotype *Salmonella*. Our results indicated that the mutant ∆*waaC12* can elicit higher cross-reactivity and can provide good protection against *S.* Choleraesuis and *S.* Enteritidis infection and that the cross-reactivity may be ascribed to an antigen of approximately 18.4–30 kDa.

## 1. Introduction

*Salmonella enterica*, which is a Gram-negative intracellular bacterial pathogen, causes clinical epidemiology hazard in humans and animals [1,2,3]. *Salmonella* can be divided into two major groups based on the disease symptoms: typhoidal *Salmonella* and non-typhoidal *Salmonella* (NTS). It has been estimated that non-typhoidal *Salmonella* causes over 93.8 million cases of gastroenteritis and even 155,000 deaths annually on a global scale [4]. In developing regions, approximately 2.5 million cases of disease with approximately 4100 deaths per year result from NTS-mediated infections, most of which are of children younger than three years, individuals with malaria or human immunodeficiency virus (HIV)-infected adults [5,6]. *S*. Typhimurium, *S*. Enteritidis and *S*. Choleraesuis account for the majority of NTS cases worldwide [7].

Vaccination is an effective strategy for the prevention of *Salmonella* infection [8]. Currently, only two *Salmonella* vaccines have been licensed for human use, both targeted against *S*. Typhi for typhoid fever [9]. While more efforts have been undertaken to develop vaccines against non-typhoidal *Salmonella*, no licensed vaccines are currently available for human use [10]. Of the vaccine candidates, whole-cell killed vaccines or polysaccharide-conjugated sub-unit vaccines are serovar-specific and induce short-term immunity, and live vaccines possess the risk of restoring virulence in immunocompromised or elderly individuals [11]. In addition, those vaccines could not confer good cross-protection against multiple-serotype *Salmonella* challenge [12].

The outer membrane, the component of the cell envelope at the outer surface of *Salmonella*, contains a lipid layer consisting of phospholipids and lipopolysaccharides and anchors substantial amounts of outer membrane proteins (OMPs) [13,14,15]. OMPs isolated from *S.* Typhimurium have proven capable of conferring protection against lethal challenge with homologous *Salmonella* in mice, indicating that OMPs are good protective antigens [16,17,18]. Previous studies have further demonstrated that purified OMPs from *Salmonella* could mediate serotype-independent protection against homologous and heterologous *Salmonella* challenges [19,20], and OMPs derived from rough mutants lacking complete lipopolysaccharide (LPS) structure and displaying the rough morphology were also able to induce cross-protective immune responses against heterologous challenge [16,18]. All of the above evidences indicate that OMPs from *Salmonella* have the potential for developing universal sub-unit vaccines to prevent homologous and heterologous *Salmonella* infections.

LPS is essential for the biosynthesis and assembly of the bacterial outer membrane [21,22]. Three different regions, including conserved lipid A, core oligosaccharide and variable *O*-antigen polysaccharide, constitute an entire LPS molecule [23]. The genes encoding the enzymes for synthesizing core oligosaccharide and *O*-antigen polysaccharide are clustered into two respective operon of waa or wba [24,25], and deletion of each gene leads to an nonreversible incomplete core or *O*-antigen of LPS [26]. For example, the mutant strain ∆*waaL* generates complete core oligosaccharide without *O*-antigen attached [26]. Truncated LPS can affect bacterial stability and outer membrane permeability, leading to the remodeling of the structure of the outer membrane and altering the outer membrane composition of the cell wall [26,27,28]. For example, the OMP profiles of *Escherichia* *coli* rough mutants were substantially distinct from the profiles of smooth strains [29,30,31]. Live *Salmonella* with truncated LPS should expose more OMPs to the host immune system, but live *Salmonella* with deep rough LPS was unable to colonize and persist in the host organs and induced poor immunogenicity against OMPs [26]. Therefore, we plan to investigate immunogenicity induced by OMPs from *S*. Typhimurium with truncated LPS of various lengths and to evaluate its cross-protection efficacy against infections with homologous and heterologous serotypes of *Salmonella*.

## 2. Results

### 2.1. Mutant Construction and Phenotype Evaluation

**Figure 1:**
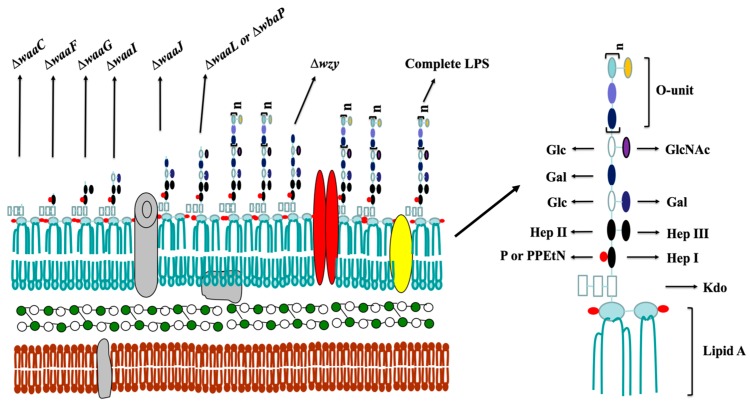
Schematic molecular model of *S.* Typhimurium cell wall structure. The *Salmonella* cell wall comprises the inner membrane, the periplasmic space filled with a gel-like matrix and the outer membrane. The inner membrane is the innermost component, whereas the outer membrane is the outermost impermeable structure, which is a bilayer consisting of a phospholipid layer on the inner side, and a lipopolysaccharide layer towards the outer side, as well as lipoproteins anchored into the membrane. Deletion of the specific gene responsible for lipopolysaccharide (LPS) synthesis would lead to truncation of LPS, thus resulting in the membrane rearrangement and altering the amounts and categories of lipoproteins. The figure represents the types of truncated LPS in this study. (Kdo, 3-deoxy-d-mannooctulosonic acid; PPEtN, pyrophosphorylethanolamine; Hep, heptose; GlcNAc, *N*-acetylglucosamine; Glc, glucose; Gal, galactose; P, phosphate). Deletion of *waaC* encoding heptosyltransferase I leads to a deep rough LPS structure carrying only Kdo-lipid A (Re); deletion of *waaF* encoding heptosyltransferase II results in a mutant generating a LPS structure with lipid A and a truncated inner core (Rd2); deletion of *waaG* encoding a glycosyltransferase leads to the Rd1 type of LPS lacking the outer core, and consequently, *waaI* and *waaJ* mutants produce the truncated core phenotype of Rb3 and Rb2, respectively; deletion of both *waaL* encoding *O*-antigen ligase and *wbaP* encoding Und-P galactose phosphotransferase leads to the production of core-lipid A (Ra) in the mutants; *wzy* encoding *O*-antigen polymerase is one of three processing genes, the deletion of which results in a mutant generating a semi-rough LPS carrying a single *O*-antigen unit with a complete lipid A core, and the deletion of *rfaH* encoding the transcriptional antiterminator leads to the production of the truncated core phenotype (Rb3).

**Figure 2:**
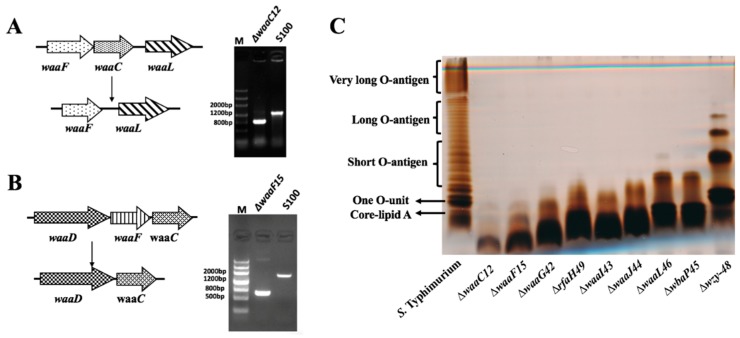
Mutant construction and LPS profiles. (**A**) Map of the deletion mutation of ∆*waaC12* (**left**), and PCR verification of the ∆*waaC12* (**right**); (**B**) map of the deletion mutation of *waaF15* (**left**) and PCR verification of the ∆*waaC12* (**right**); and (**C**) LPS patterns of the mutants and the parent strain. LPS was visualized by silver straining on polyacrylamide gel electrophoresis (PAGE) gels. The expected location of *O*-antigen components and the core is shown on the left, and the *O*-unit is a repeating unit of the *O*-antigen.

We used our published methods to construct a series of *S.* Typhimurium mutant strains using the parent strain S100, which was isolated from a duck infected with *Salmonella* [26] (Figure 1 and Figure 2). We also constructed the additional mutants ∆*waaC12* and ∆*waaF15*, and the PCR results confirmed that the deletions were successful (Figure 2A,B).

The LPS profile results showed that the ∆*waaC12* and ∆*waaF15* mutants had shorter lengths of LPS than the other mutants constructed in the previous study (Figure 2C) [26]. The LPS generated by the ∆*waaC12* mutant contained lipid A and 3-deoxy-d-mannooctulosonic acid (Kdo) moieties and migrated faster than the LPS generated by the ∆*waaF15* mutant and other mutants, including ∆*waaG42* to ∆*wzy-48*; LPS produced by the ∆*waaF15* mutant included lipid A, Kdo and one heptose moiety (Figure 2C). The mutants displayed distinct LPS lengths, from one *O*-antigen unit in the ∆*wzy-48* mutant to only Kdo in the ∆*waaC12* mutant.

### 2.2. Preparation of OMPs

**Figure 3:**
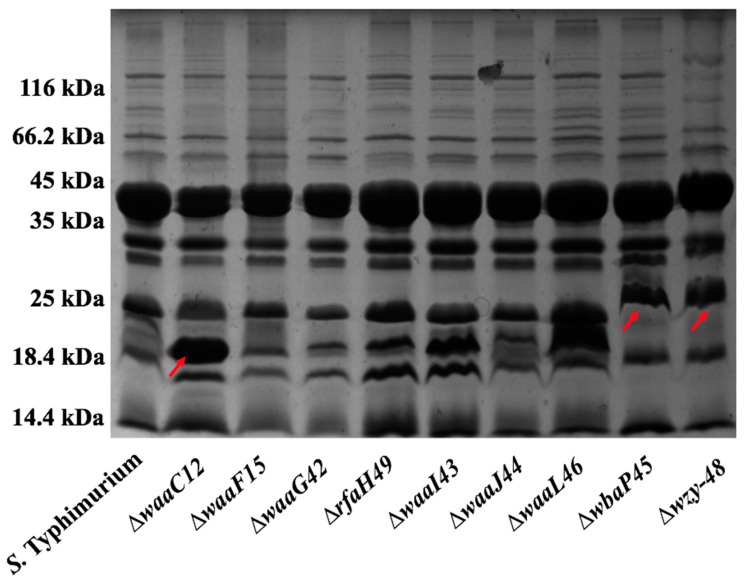
Outer membrane proteins’ (OMPs) profile. OMPs purified from the parent strain and its mutants were separated by sodium dodecyl sulfate polyacrylamide gel electrophoresis (SDS-PAGE) on 12% gel and subjected to Coomassie brilliant blue staining. The mutant strains (from left to right): S100 (wild-type strain), S511 (∆*waaC12*), S512 (∆*waaF15*), S372 (∆*waaG42*), S377 (∆*rfaH49*), S373 (∆*waaI43*), S374 (∆*waaJ44*), S376 (∆*waaL46*), S375 (∆*wbaP45*), S378 (∆*wzy-48*). The red arrows represented the bands of differential proteins.

As shown in Figure 3, the majority of proteins in the OMP profiles were between 35 and 45 kilodaltons (kDa), corresponding to the major porins, including OmpA, OmpC and OmpD [32,33]. The protein bands of low molecular weights ranging from 18.4 to 35 kDa presented a slight divergence between the mutants and the wild-type strain (Figure 3). For instance, one up-regulated expression of the protein, the size of which was approximately 18.4 kDa, was observed in the ∆*waaC12* mutant, and one just above the 25-kDa protein band was present in ∆*wbaP45* and ∆*wzy-48* mutants (red arrows). This result indicated that the genetic modification associated with the synthesis of LPS might result in the up- or down-regulated expression of diverse minor proteins by affecting the stability and permeability of the outer membrane [26,27,28].

### 2.3. Evaluation of the Immunogenicity of OMPs from Wild-Type and Truncated LPS Salmonella

**Figure 4:**
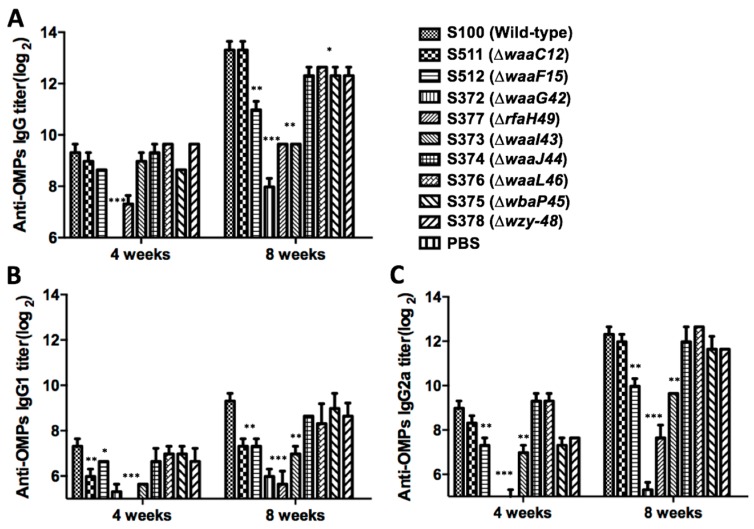
Serum antibody responses in mice immunized by the intranasal route. Total serum IgG specific for OMPs from *S.* Typhimurium (**A**), IgG1 specific for OMPs from *S.* Typhimurium (**B**) and IgG2a specific for OMPs from *S.* Typhimurium (**C**) were measured by enzyme-linked immunosorbent assay (ELISA). Each group has eight mice. The data represented the reciprocal anti-IgG antibody titer in pooled sera from mice immunized with OMPs from parent strain S100 and its mutants with truncated core or *O*-antigen at the indicated weeks after immunization. The error bars represented variations between triplicate wells. The mice were boosted at Week 5. *** *p* < 0.001; ** *p* < 0.01; * *p* < 0.05; compared to titers from mice immunized with OMPs isolated from wild-type strain S100. No immune responses were detected to OMPs in mice immunized with PBS (reciprocal titer < 1:50). Immune responses (IgG, IgG1 and IgG2a) against OMPs from *S.* Typhimurium were below the detection level in the serum from mice immunized with OMPs from mutants ∆*waaG* and ∆*rfaH* at four weeks (reciprocal titer < 1:50).

To evaluate the immunogenicity induced by OMPs from diverse truncated LPS *Salmonella*, 10 groups of mice were immunized intranasally using purified OMPs from nine mutants and the wild-type S100 strain with 10 μg OMPs in 5 μL HEPES buffer and were then boosted with the same dose of the corresponding OMPs five weeks later. We measured the serum IgG, IgG1 and IgG2a antibody responses against OMPs from the wild-type S100 strain four and eight weeks after the first immunization (Figure 4). As shown in Figure 4, the OMPs from the wild-type S100 strain induced the highest level of IgG among all of the groups, while the OMPs from the ∆*waaG42* mutant elicited the lowest IgG titer, significantly lower than the titers for the other groups (*p* < 0.01 to 0.001). At eight weeks (three weeks after the booster immunization), the ∆*waaJ44*, ∆*waaL46*, ∆*wbaP45* and ∆*wzy-48-*induced anti-OMP IgG titers were similar to each other and significantly lower than the titer for the wild-type strain (*p* < 0.05). Interestingly, the titer of anti-OMP IgG induced by OMPs from the mutant ∆*waaC12*, possessing the simplest structure of the “deep rough” phenotype LPS, was similar to that of the wild-type strain, with no significant difference.

We also determined the levels of anti-OMP IgG isotype subclasses IgG1 and IgG2a of serum from the mice of each group. As shown in Figure 4B,C, the IgG1 titers were similar to the IgG2a levels in the early phase (four weeks), but the IgG2a titers of all groups increased more rapidly than the IgG1 titers at eight weeks, indicating that the immune response exhibited a shift to a Th1-biased type. All groups of OMPs showed similar IgG1/IgG2a ratios, indicating that they elicited balanced Th1/Th2 responses. OMPs from the ∆*waaF15*, ∆*waaG42*, ∆*rfaH49* and ∆*waaI43* groups showed poor immunogenicity, and the titers of IgG1 and IgG2a were lower than for the other groups at four and eight weeks (*p* < 0.05 to 0.001).

### 2.4. Evaluation of Protection against Challenge by the S. Typhimurium Wild-Type Strain

To investigate the effects of OMPs from LPS mutants on the protection efficacy, BALB/c mice were challenged via the oral route with 1 × 10^9^ CFU of *S.* Typhimurium strain S100 at five weeks after the booster immunization. Immunization with OMPs of ∆*waaC12*, ∆*waaJ44*, ∆*waaL46*, ∆*wbaP45* and ∆*wzy-48* induced greater protective immunity than the other groups (Table 1), and the group with OMPs of the wild-type strain S100 showed 100% protection against homologous *Salmonella* challenge. The mice of the ∆*waaF15*, ∆*waaG42*, ∆*rfaH49* and ∆*waaI43* groups showed weak protection against *Salmonella* infection, and survival rates ranged from 25% to 50%, indicating that the OMPs from those mutants exhibited less protective immunity. All of the mice of the control group (PBS group) succumbed to *S.* Typhimurium strain S100 infection.

### 2.5. Evaluation of Cross-Reactivity with OMPs from Heterologous Serotype Salmonella

**Figure 5:**
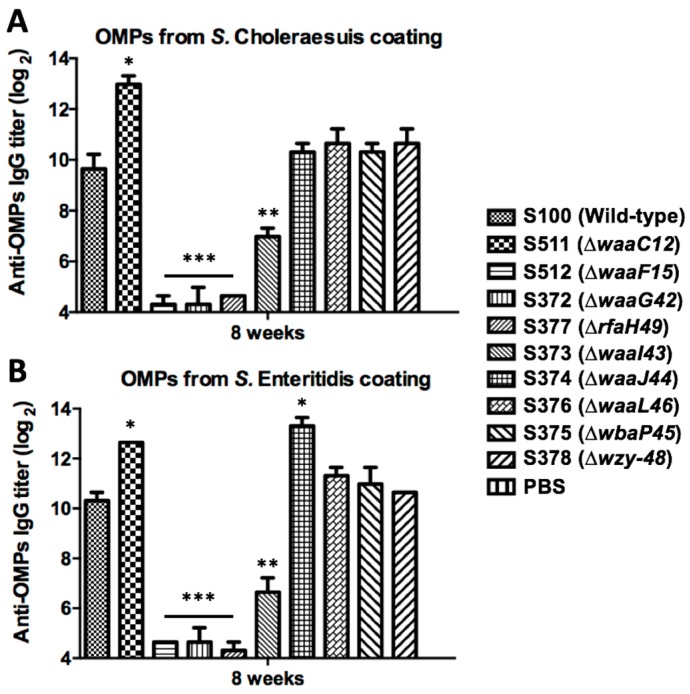
IgG cross-reactivity against OMPs from other *Salmonella* serogroups. The sera were obtained from mice (*n* = 8) immunized with OMPs from the parent strain and its mutants with the truncated core or *O*-antigen at eight weeks after the primary immunization and were pooled to evaluate IgG cross-reaction against OMPs isolated from heterologous diverse *Salmonella*, including *S.* Choleraesuis (**A**) and *S.* Enteritidis (**B**). The error bars represent variations from triplicate wells. *** *p* < 0.001; ** *p* < 0.01; * *p* < 0.05l; compared to titers from mice immunized with OMPs from parent strain S100.

To evaluate the cross-reactivity of serum antibodies from the mice immunized using OMPs of wild-type and truncated LPS *Salmonella* mutants, we detected the antibody responses of pooled serum from mice immunized with OMPs isolated from all mutant strains against purified OMPs from multiple *Salmonella* strains, including *S.* Choleraesuis and *S.* Enteritidis. The IgG levels of the ∆*waaC12* groups were higher than the levels in the parent strain group against the OMPs from *S.* Choleraesuis and *S.* Enteritidis, and both showed significant differences compared to the parent strain (*p* < 0.05) (Figure 5). The ∆*waaJ44*, ∆*waaL46*, ∆*wbaP45* and ∆*wzy-48* groups also elicited higher reactivities than the wild-type S100 group against OMPs isolated from *S.* Choleraesuis and *S.* Enteritidis, and only the ∆*waaJ44* group showed a significant difference compared to the S100 group (*p* < 0.05).

**Figure 6:**
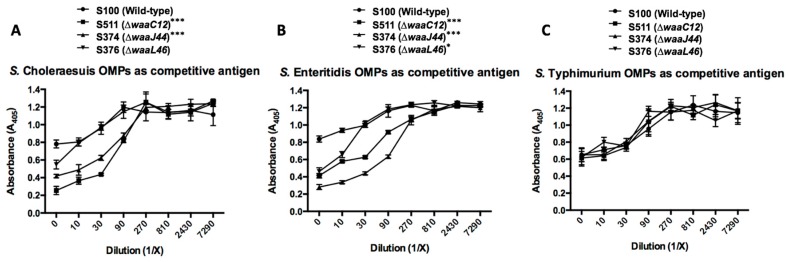
Competitive ELISA to confirm the cross-reactivity of OMPs from the truncated LPS mutants against heterologous *Salmonella*. OMPs isolated from *S.* Typhimurium were incubated in the plate as the coating antigen. OMPs from *S.* Choleraesuis (**A**) or *S.* Enteritidis (**B**) as the competitive antigen and OMPs from *S.* Typhimurium (**C**) as the control competitive antigen diluted from 1/10 to 1/7290 (Dilution, 1/X) were incubated in wells. The sera were obtained from mice (*n* = 8) immunized with OMPs from the parent strain and its mutants at eight weeks after the primary immunization. The error bars represent variations from triplicate wells. *** *p* < 0.001; * *p* < 0.05; compared to titers from mice immunized with OMPs from the parent strain S100.

Furthermore, the competitive ELISA was used to confirm the cross-reactivity of OMPs from the wild-type and the truncated LPS mutant strains. The ∆*waaC12* and ∆*waaJ44* mutants elicited significant cross-reactivity of *S.* Typhimurium OMPs against OMPs from *S.* Choleraesuis (*p* < 0.001); however, the ∆*waaL46* group had no significant difference of cross-reactivity compared to the wild-type group (Figure 6A). OMPs from all three mutants presented the induction of cross-reactivity against OMPs from *S.* Enteritidis (*p* < 0.001 or 0.05) (Figure 6B). The competitive ELISA results further proved the increased cross-reactivity of OMPs from truncated LPS mutants against heterologous *Salmonella*.

### 2.6. Evaluation of Cross-Protection against Heterologous Serotype Salmonella

**Figure 7:**
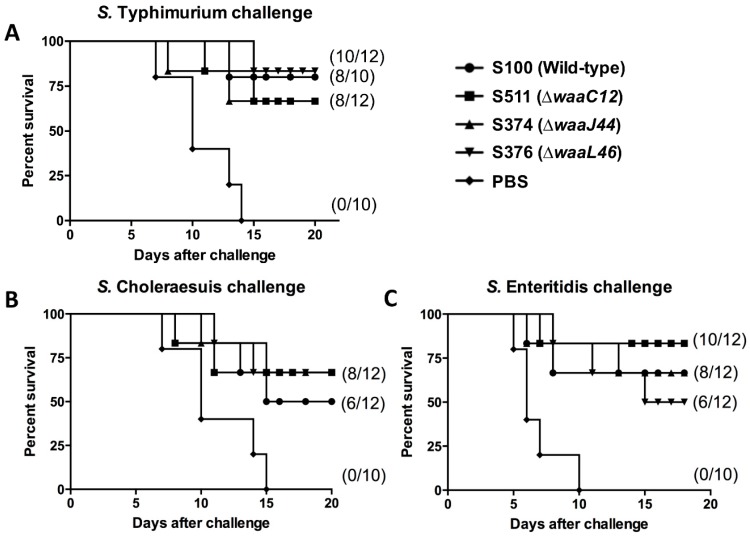
Cross-protective efficacy induced by OMPs. Ten (control) or 10 to 12 (vaccine) BALB/c mice per group were intranasally immunized twice at four-week intervals with OMPs isolated from the indicated Salmonella mutants. The immunized mice were orally challenged with 10^9^, 10^7^ and 10^7^ CFU of S100 (*S.* Typhimurium) (**A**), S340 (*S.* Choleraesuis) (**B**) and S246 (*S.* Enteritidis) (**C**), respectively, at eight weeks after the first immunization. Mortality was monitored for three weeks after challenge. The data were combined from the two separate experiments, which showed similar results, and the numbers in parentheses referred to the number of surviving mice and the total number of mice per group. All vaccine groups were significantly different from the PBS control (*p* < 0.01). There was no significant difference in protection among the other groups.

To investigate the protection efficacy against challenges by different *Salmonella* serotypes, immunized mice of twelve groups, including nine experimental groups and three control groups, were independently challenged via oral administration of 10^9^ (10^4^ × LD_50_), 10^7^ (~10^2^ × LD_50_) or 10^7^ (~10^2^ × LD_50_) CFU of S100 (*S.* Typhimurium), S246 (*S.* Enteritidis) or S340 (*S.* Choleraesuis), respectively. This experiment was performed twice. The results from the two experiments were similar and were pooled for analysis. As shown in Figure 7A, the protection rate against *S.* Typhimurium was consistent with the previous results (Table 1), and OMPs isolated from the wild-type S100 strain induced a strong protective immunity against *S.* Typhimurium. Immunization with OMPs from the ∆*waaC12*, ∆*waaJ44* and ∆*waaL46* mutants provided 66.7% (8/12) protection for mice after *S.* Choleraesuis challenge, and they could induce higher protection efficacy than immunization with OMPs from the parent strain S100 (Figure 7B) (*p* = 0.46). For *S.* Enteritidis (Figure 7C), the degrees of protection provided among various groups were different. The best protection levels were provided by the ∆*waaC12*, which elicited 83% (10/12) survival and exhibited higher protection than the wild-type strain. Interestingly, the ∆*waaL46* group induced lower protection (50%, 6/12) than the S100 group (66%, 8/12), indicating that the protection efficacy of the OMPs isolated from the mutant ∆*waaL46* was not consistent with the immunogenic capability (Figure 7).

## 3. Discussion

Due to the emergence of multiple drug resistance in *Salmonella*, Salmonellosis is currently difficult to control [34,35,36]. Vaccination is a powerful tool for the control of this disease. Traditional subunit vaccines consisting of single protein antigens have limited vaccine efficacy and protection due to antigenic variation among *Salmonella* strains [8]. Therefore, vaccines containing OMPs capable of inducing a cross-protective immune response against multiple serotypes of *Salmonella* may be a good option for developing vaccines against multiple *Salmonella* serovars. In our previous study, we demonstrated that *Salmonella* mutants carrying truncated LPS were not good live vaccines, as they failed to induce strong immune responses and cross-reactivity to other enteric bacteria [26]. In this study, we systematically investigated the immunogenicity and cross-protection efficiency of OMPs purified from mutants, including ∆*waaC12*, ∆*waaF15*, ∆*waaG42*, ∆*rfaH49*, ∆*waaI43*, ∆*waaJ44*, ∆*waaL46*, ∆*wbaP45* and ∆*wzy-48*, and discovered potential OMP vaccines to protect against homologous or heterologous serotype *Salmonella* infection.

In this study, we used the method of detergent-insoluble isolation for OMPs preparation, and this method was the initial extraction of the outer membrane complex, composed of a large amount of OMPs, a small amount of LPS and other cell membrane components [37,38]. Additionally, OMPs purified from several Gram-negative bacteria following this method were commonly used to evaluate its potential as subunit vaccines [39,40]; therefore, we consider this outer membrane complex to be OMPs.

It has been demonstrated that LPS structure can affect the outer membrane compositions of bacteria [27,28]. Based on examples from *E. coli* studies*,* the OMP profiles of *E*. *coli* rough mutants were substantially distinct from the profiles of smooth strains [29,30,31]. In this study, differences in the OMP profiles of mutants with truncated and smooth LPS were also observed, but it seems that truncated LPS did not significantly influence the composition of the OMPs (Figure 3).

OMPs from different *Salmonella* and parent strains were administered to the mice via intraperitoneal and intranasal routes. Vaccination by the intraperitoneal route caused severe symptoms in mice, which might result from toxic lipid A [41,42]; therefore, we did not show those results in this study. The purpose of this study was to investigate the immunogenicity and protection efficacy conferred by OMPs from *S*. Typhimurium mutants with diverse truncated LPS and to discover the best OMPs for developing a universal subunit vaccine for multiple *Salmonella* infection. We selected three *Salmonella* serotypes to evaluate the immune responses and challenges, including *S.* Typhimurium, belonging to the B1 serotype group, which is the natural strain infecting mice and other animals, and was used for OMP preparation [43]; *S*. Enteritidis, belonging to the D1 serotype group, whose natural host is chicken and poultry [44,45]; and *S*. Choleraesuis, belonging to the C1 serotype group, whose natural host is pig [46,47]. These three *Salmonella* serotypes contribute to the majority of non-typhoidal *Salmonella* (NTS) cases worldwide [6], and they are able to establish systemic infection in mice after oral administration. Therefore, we selected the mouse model to determine the cross-protection conferred by OMPs from various LPS mutants after *S.* Typhimurium, *S.* Choleraesuis and *S.* Enteritidis infection.

The immune responses induced in mice by OMPs from various LPS mutants were diverse, showing that OMPs isolated from wild-type strain *Salmonella* could induce the highest antibody levels against OMPs, and OMPs from the ∆*waaG42* mutant induced the least antibody levels against OMPs from *S.* Typhimurium (Figure 4 and Figure 5). IgG antibodies from mice immunized with OMPs from ∆*waaC12* and ∆*waaJ44* mutants were significantly more reactive against OMPs from *S.* Choleraesuis and *S.* Enteritidis than IgG antibodies from mice immunized with wild-type OMPs, and IgG antibodies from mice immunized with OMPs from ∆*waaL46*, ∆*wbaP45* and ∆*wzy-48* mutants showed the same reactions against OMPs from *S.* Choleraesuis and *S.* Enteritidis as IgG antibodies from mice immunized with OMPs from the wild-type strain (Figure 5), indicating that the *O*-antigen polysaccharide in OMPs from the wild-type *S.* Typhimurium will also contribute to the induction of immune responses, which was also supported by the protection rate after challenge with wild-type *S.* Typhimurium, showing that OMPs from wild-type *S.* Typhimurium conferred a 100% protection rate, while OMPs from other mutants conferred lower protection efficacies (Table 1). The OMPs isolated from ∆*waaL46* and ∆*wbaP45* mutants generating the same LPS moiety of lipid A with the entire core did not provide the same protection rate against wild-type *S*. Typhimurium challenge (Table 1), indicating that protection capacity provided by OMPs from truncated LPS mutants may primarily be ascribed to induced immunity to OMPs, not to truncated LPS [48].

Based on the results obtained from the first animal experiments, we selected OMPs from the mutants ∆*waaC12*, ∆*waaJ44* and ∆*waaL46* to evaluate their capacity to induce cross-protection against multiple *Salmonella* infections. As immunized OMPs purified from *Salmonella* and its mutant derivatives would be contaminated by small amounts of various truncated LPS possessed by *Salmonella* species, these conserved oligosaccharides might only confer limited cross-protection compared to conserved OMPs during the smooth *Salmonella* infection [48]. For examples, in our study, *S*. Typhimurium and *S*. Enteritidis share O1 and O12 *O*-antigen epitopes in their *O*-antigen polysaccharide [49,50]; OMPs purified from wild-type *S*. Typhimurium, which was contaminated by wild-type LPS carrying O1 and O12 epitopes, did not provide better protection against *S*. Enteritidis infection than OMPs from ∆*waaC12* and ∆*waaJ44* mutants (Figure 7). Oligosaccharides isolated from rough *S.* Gallinarum 9R, which, coupled to bovine serum albumin (BSA) directly, induced cross-reactivity between serum and heterologous smooth LPS of *S*. Enteritidis, but did not provide protection against lethal challenge of *S*. Enteritidis [51], indicated that truncated LPS in vaccines plays minor roles to protect against the pathogenic smooth LPS *Salmonella* infection; the evidence supporting our notion also came from earlier studies in that antibodies to the Rc or Re core of LPS only provided a limited spectrum of protection, as these antisera only have a limited ability to react with smooth LPS [52,53,54].

We observed that OMPs from the ∆*waaC12* mutant were able to promote cross-reaction and to provide good protection against *S.* Choleraesuis and *S.* Enteritidis infections (Figure 5, Figure 6 and Figure 7). This cross-protection conferred by the crude OMPs from the *waaC* mutant would contribute primarily to the cross-protective OMPs, where contamination of lipid A with Kdo in OMPs may function as an optimal adjuvant and enhanced the immune responses to OMP antigens [41,42]; the components of OMPs from *waaC* mutant serving as cross-protective antigens also need to be determined in a future study.

In summary, we systematically investigated the immunogenicity and protective efficacy of crude OMPs isolated from truncated LPS *Salmonella* mutant strains and found that OMPs from the mutant ∆*waaC12* in our tested OMPs could elicit enhanced cross-protective immunity against heterologous serotype *Salmonella*.

## 4. Materials and Methods

### 4.1. Bacterial Strains, Plasmids, Media and Growth Conditions

All strains and plasmids associated with this study are listed in Table 2. *Salmonella* Typhimurium (*S.* Typhimurium) and *Escherichia coli* (*E*. *coli*) were grown in Luria–Bertani (LB) [55] broth or agar or nutrient broth (Difco, Detroit, MI, USA) at 37 °C. The growth of ∆*asd* strains needed to be added to 50 μg/mL of diaminopimelic acid (DAP) [56]. Sucrose-LB agar (5% concentration) was used for *sacB* gene-based counter selection in mutant strains’ construction.

### 4.2. Construction of Plasmids and Mutations

The primers designed in this study are listed in Table S1. The protocol of DNA manipulations was performed as previous described [58]. Transformation of *E*. *coli* was done by electroporation. Positive clones were selected on LB agar plates containing appropriate antibiotics. Asd^+^ plasmids were selected by LB agar plates.

We followed the published methods to construct *Salmonella* mutants from ∆*waaG42* to ∆*wzy-48* based on parent *S.* Typhimurium S100 [26]. For construction of the ∆*waaC* mutation, two pairs of primers, waaC-1F/waaC-1R and waaC-2F/waaC-2R, were used for the *waaC* deletion and used to amplify approximately 350-bp upstream and downstream fragments of gene *waaC*, respectively, from the S100 (*S.* Typhimurium); then, two fragments were joined by PCR using primers waaC-1F/waac-2R. The resulting PCR product was added to the terminal A by using the LA Taq enzyme (Takara, Dalian, China) and then was co-incubated with the T-terminal pYA4278 vector, which was digested by AhdI enzyme, resulting in plasmid pQK256. The construction of pQK257 for *waaF* gene deletion used the same strategy as *waaC* gene deletion.

∆*waaC12* and ∆*waaF15* mutations were independently introduced into *S.* Typhimurium by the suicide plasmid method [59]. Briefly, S100 (*S.* Typhimurium) bacterial cultures were conjugated with *E. coli* χ7213 harboring suicide plasmids pQK256 (for ∆*waaC*) or pQK257 (∆*waaF*) as the ratio of 1:1.5 on the LB agar plate. After 24 h of incubation, the bacteria cell complexes were scratched and streaked on the LB agar plate containing 25 μg/mL chloramphenicol (Cm); the next day, the single colony was picked up and grown on the plates with Cm antibiotic, and the single crossover strains was grown in the fresh LB for 4 h, was diluted to LB by a suitable ratio and spread on the LB plates containing 5% sucrose overnight at room temperature. The LB plate and LB plate containing Cm antibiotic were used to screen the positive colony; the colony that cannot grow on the LB plate containing Cm antibiotic will be identified by colony PCR. The other mutation ∆*waaF* could be introduced into S100 to generate the mutant S512 by the same strategy as mutant ∆*waaC*.

### 4.3. The Lipopolysaccharide (LPS) Profile of Salmonella Mutant Strains

LPS profiles were determined by the previous method as described [60]. Briefly, 2 mL of overnight cell culture were pelleted and resuspended in 150 μL of Dissociation Buffer A (0.5 M Tris-Cl pH 6.8, 10% glycerol, 10% SDS, 5% β-mercaptoethanol); then, the samples were boiled for 10 min. After cooling at room temperature, the samples were centrifuged for 15 min. The supernatant was diluted 1:10 (10 into 90 μL) into Dissociation Buffer B (0.5 M Tris-Cl pH 6.8, 10% glycerol, 0.05% bromophenol blue), and 1 μL 20 mg/mL proteinase K (Sigma-Aldrich, St. Louis, MO, USA) was added into the dilution and incubated at 37 °C for 1 h. After that, the samples were separated on 12% gels (sodium dodecyl sulfate polyacrylamide gel electrophoresis (SDS-PAGE)) and stained by silver staining.

### 4.4. Outer Membrane Proteins (OMPs) Purification

The method of preparation of OMPs was based on the method used by Carlone and Thomas [37]. Briefly, 500 mL of overnight culture were harvested and resuspended in 25 mL of 10 mM HEPES buffer (pH 7.4) on ice. The cells were lysed by FRENCH Pressure (Thermo IEC, Needham Heights, MA, USA); the samples were centrifuged (15,600× *g*, 2 min, 4 °C) for removing the unbroken cells; the supernatants were transferred into a new tube and centrifuged (15,600× *g*, 30 min, 4 °C). The pellets were then resuspended in 10 mL 10 mM HEPES buffer (pH 7.4); 10 mL 2% sarkosyl was added into the supernatant and mixed well and then incubated for 30 min at room temperature with gentle shaking on a vortex Genie. After centrifugation (15,600× *g*, 30 min, 4 °C), the pellets were washed once by 20 mL 10 mM HEPES buffer (pH 7.4) and finally resuspended in 1 mL Phosphate Buffered Saline (PBS) buffer (pH 7.4). Purified OMPs were stored at −80 °C.

The concentrations of OMPs were measured by the BCA protein assay kit (Thermo Pierce, Rockford, IL, USA), and 10 μg OMPs were analyzed by SDS-PAGE and strained by Coomassie brilliant blue (Bio-Rad, Richmond, CA, USA).

### 4.5. Animal Experiments

Animal research was carried out in accordance with the Animal Welfare Act and regulations related to animal experiments (Ya’an, China; Approval No. 2011-028). The principle stated in the Guide for the Care and Use of Laboratory Animals was followed. The animal care protocols were approved by Sichuan Agricultural University. All efforts were made to minimize animal suffering during the experiments.

BALB/c mice of 6 weeks of age were used for all of the animal experiments and purchased from the Dashuo Biotechnology Co., Ltd. (Chengdu, China). Eight to twelve mice for each group were immunized intranasally on Day 0 and boosted at Week 5 with 10 μg OMPs in 5 μL PBS buffer per mice, and 5 μL PBS buffer served as the control. The blood samples were collected at the interval of two weeks after the immunization.

To determine immune response induced by OMPs isolated from *Salmonella* strains, including ∆*waaC12*, ∆*waaF15*, ∆*waaG42*, ∆*rfaH49*, ∆*waaI43*, ∆*waaJ44*, ∆*waaL46*, ∆*wbaP45*, ∆*wzy-48* and S100, the mice of eleven groups were immunized by intranasal route with OMPs and the negative control. Five weeks after the booster immunization, the mice were challenged with 10^9^ colony-forming units (CFU) of S100 (*S.* Typhimurium) in 20 μL buffered saline with 0.01% gelatin (BSG) by the oral route for determining the protection rates. Fifty percent lethal death (LD_50_) of S100 is about 10^5^ CFU using the mouse model. The mice were monitored daily for 30 days after challenge.

To evaluate the cross-protective ability conferred by immunization of OMPs from diverse *Salmonella* strains, we selected three *Salmonella* mutants for the animal experiment, including ∆*waaC12*, ∆*waaJ44* and ∆*waaL46*, the OMPs of which provided better protection efficacy against the challenge of *S.* Typhimurium and immune responses against OMPs from *S.* Typhimurium, *S.* Enteritidis and *S.* Choleraesuis. Five weeks after the booster immunization, the mice were challenged with 10^9^, 10^7^ and 10^7^ CFU of S100 (*S.* Typhimurium), S246 (*S.* Enteritidis) and S340 (*S.* Choleraesuis) in 20 μL BSG by the oral route, respectively. The challenged mice were monitored daily for 30 days.

### 4.6. Analysis of Antibody Response in Mice

The enzyme-linked immunosorbent assay (ELISA) method was used to analyze the antibody responses induced by OMPs [61]. Briefly, 2 μg of OMPs from *S.* Typhimurium (S100), *S.* Enteritidis (S246) or *S.* Choleraesuis (S340) in 100 μL sodium carbonate bicarbonate coating buffer (pH 9.6) were used to coat each well of the 96-well plates and then incubated overnight at 4 °C. The plate was washed 3 times with PBST (PBS with 0.1% Tween 20) and then blocked with 2% bovine serum albumin (BSA) (Sigma-Aldrich) solution. After incubation of 2 h at room temperature, a 100-μL volume of serially-diluted sample (serum dilution started at 1:50) was added to triplicate individual wells and incubated for 1 h at room temperature and then washing with PBST 3 times; biotinylated goat anti-mouse IgG, IgG1 or IgG2a (Southern Biotechnology Inc., Birmingham, AL, USA) was added in each well, and then, the wells were developed with a streptavidin-alkaline phosphatase conjugate (Southern Biotechnology, Inc., Birmingham, AL, USA) and detected using p-nitrophenylphosphate substrate (Sigma-Aldrich) in diethanolamine buffer (pH 9.8). Absorbance was taken at 405 nm using an automated ELISA plate (Bio-Rad iMark Microplate Reader, Hercules, CA, USA) with suitable time. The data A405, which had 0.1 higher than the PBS control values, was considered positive.

The competitive ELISA of serum IgG antibody between OMPs from *S.* Typhimurium and *S.* Enteritidis or *S.* Choleraesuis was used to confirm the cross-reactivity of the OMPs from different LPS mutants conferring strong immunity and good protective efficacy [62]. Two micrograms per well of the OMPs isolated from *S.* Typhimurium were coated in plates, and a 100-μL volume of a 50-fold diluted serum was added to the wells in triplicate. After 1 h of incubation at room temperature, OMPs from *S.* Enteritidis or *S.* Choleraesuis as the competitive antigen and OMPs from *S.* Typhimurium as the control competitive antigen diluted in several dilutions from 10-fold to 7290-fold were incubated in each well for 2 h at 37 °C. The absorbance at 405 nm was read in an automated ELISA plate (Bio-Rad iMark Microplate Reader, USA).

### 4.7. Statistical Analysis

All of the experiments were conducted in triplicate, and statistical analyses were performed using the GraphPad Prism 5 software package (Graph Software, San Diego, CA, USA) [63]. One-way analysis of variance or two-way analysis of variance (ANOVA) was used to analyze the statistical significance of the differences between mean values for various vaccinated groups and the control group. Numerical data were expressed as means ± standard deviation. The Kaplan–Meier method and log-rank test were used for the statistics and analysis of the survival curves. *p* < 0.05 was considered a significant difference; *p* < 0.01 was considered a standard significant difference; and *p* < 0.001 was considered an extremely significant difference.

## Figures and Tables

**Table 1:** Immunization with OMPs protected mice against oral challenge with *S.* Typhimurium strain S100.

Groups	No. of Surviving Mice/Total No. of Mice
S100 (wild-type)	8/8 (100%)
S511 (∆*waaC12*)	6/8 (75%)
S512 (∆*waaF15*)	4/8 (50%)
S372 (∆*waaG42*)	2/8 (25%)
S377 (∆*rfaH49*)	2/8 (25%)
S373 (∆*waaI43*)	4/8 (50%)
S374 (∆*waaJ44*)	6/8 (75%)
S376 (∆*waaL46*)	8/8 (100%)
S375 (∆*wbaP45*)	6/8 (75%)
S378 (∆*wzy-48*)	6/8 (75%)
Phosphate Buffered Saline (PBS)	0/8 (0%)

**Table 2:** Bacterial strains and plasmids used in this study.

Strains or Plasmids	Description	Source
Strains		
S100	*S.* Typhimurium, clinical isolate from duck	IPVM *
S511	∆*waaC12*	This work
S512	∆*waaF15*	This work
S372	∆*waaG42*	[26]
S377	∆*rfaH49*	[26]
S373	∆*waaI43*	[26]
S374	∆*waaJ44*	[26]
S376	∆*waaL46*	[26]
S375	∆*wbaP45*	[26]
S378	∆*wzy-48*	[26]
S246	*S.* Enteritidis, clinical isolate from chicken	IPVM
S340	*S.* Choleraesuis, clinical isolate from pig	IPVM
*E. coli*	*–*	–
χ7232	*endA1 hsdR17 (rK-,mK+) supE44 thi-1 recA1 gyrArelA1Δ (lacZYA-argF) U169*λpir*deoR* (φ*80dlac Δ (lacZ) M15*)	[57]
χ7213	thi-1 thr-1 leuB6 glnV44 tonA21 lacY1 recA1 RP4-2-Tc::μλpir Δ*asdA4* Δ*zhf-2* ::Tn 10	[57]
**Plasmids**		
pYA4278	Suicide plasmid (pRE112)	[26]
pQK256	For deletion of *rfaC*	This work
pQK257	For deletion of *rfaF*	This work

* IPVM: Institute of Preventive Veterinary Medicine at the Sichuan Agricultural University in China.

## Supplementary Materials

Supplementary materials can be found at http://www.mdpi.com/1422-0067/17/3/416/s1.
