# Supplementary material for: Immunogenicity and Cross-Protective Efficacy Induced by Outer Membrane Proteins from Salmonella Typhimurium Mutants with Truncated LPS in Mice
Source: Int J Mol Sci. 2016 Mar 22;17(3):416. doi: 10.3390/ijms17030416 (PMC4813267; doi:10.3390/ijms17030416)
Supplement: Supplementary file 1 [file ijms-17-00416-s001.pdf]

Supplementary Materials: Immunogenicity and Cross-Protective Efficacy Induced by Outer Membrane Proteins from *Salmonella* Typhimurium Mutants with Truncated LPS in Mice

Qiong Liu, Qing Liu, Xinxin Zhao, Tian Liu, Jie Yi, Kang Liang and Qingke Kong

Table S1. Primers used in this study.

Primers	Sequences (5′-3′)	Function
rfaC-1F	TGGCGGCGCTGAATAGCGAG	For deletion of *waaC* (*rfaC*) by suicide plasmid
rfaC-1R	CTCTTTACCTGCAGGTTAAACGCCCTCTTCCGACAAC	
rfaC-2F	GTTTAACCTGCAGGTAAAGAGACTCTGTCTCATCCCA	
rfaC-2R	TAAGAAGCCCTCCAGTACCG	
rfaF-1F	GAATACACAGTTAAACAACGG	For deletion of *waaF* (*rfaF*) by suicide plasmid
rfaF-1R	CCCTCTCCTGCAGGACTTACGCGTCGGTTCAGC	
rfaF-2F	GTAAGTCCTGCAGGAGAGGGCGTTTAATGCGGGTT	
rfaF-2R	TTTATCCCATGCGCCAGACG	
